# Proteomic Analysis of Tung Tree (*Vernicia fordii*) Oilseeds during the Developmental Stages

**DOI:** 10.3390/molecules21111486

**Published:** 2016-11-08

**Authors:** Zhiyong Zhan, Yicun Chen, Jay Shockey, Xiaojiao Han, Yangdong Wang

**Affiliations:** 1 State Key Laboratory of Tree Genetics and Breeding, Chinese Academy of Forestry, Beijing 100091, China; zhanzhiyong862007@163.com (Z.Z.) yicun_chen@163.com (Y.C.); hanxiaojiao1004@163.com (X.H.); 2 Research Institute of Subtropical Foresty, Chinese Academy of Forestry, Fuyang 311400, Zhejiang, China; 3 Jiangxi Academy of Forestry, Nanchang 330013, Jiangxi, China; 4 United States Department of Agriculture-Agricultural Research Service, Southern Regional Research Center, New Orleans, LA 70124, USA; jay.shockey@ars.usda.gov

**Keywords:** tung tree, seed, oil content, oil body, development, proteomics

## Abstract

The tung tree (*Vernicia fordii*), a non-model woody plant belonging to the Euphorbiaceae family, is a promising economic plant due to the high content of a novel high-value oil in its seeds. Many metabolic pathways are active during seed development. Oil (triacylglycerols (TAGs)) accumulates in oil bodies distributed in the endosperm cells of tung tree seeds. The relationship between oil bodies and oil content during tung tree seed development was analyzed using ultrastructural observations, which confirmed that oil accumulation was correlated with the volumes and numbers of oil bodies in the endosperm cells during three different developmental stages. For a deeper understanding of seed development, we carried out proteomic analyses. At least 144 proteins were differentially expressed during three different developmental stages. A total of 76 proteins were successfully identified using matrix-assisted laser desorption/ionization time-of-flight mass spectrometry/mass spectrometry (MALDI-TOF/MS/MS). These proteins were grouped into 11 classes according to their functions. The major groups of differentially expressed proteins were associated with energy metabolism (25%), fatty acid metabolism (15.79%) and defense (14.47%). These results strongly suggested that a very high percentage of gene expression in seed development is dedicated to the synthesis and accumulation of TAGs.

## 1. Introduction

Seed development and germination have been studied using transcriptome and proteomic analyses, either independently or in combination [1], and these methods have had an increased impact on the study of the seed proteome [2]. Most of the papers related to seed proteomics have focused on the construction of reference proteome maps at specific stages [3,4] including dormancy [5], germination [2,6,7], post-germination [8,9], development and maturation [10,11]. During this period, comparative proteomics was used as a basic method to provide insight into the identification of functional gene products and their expression levels in seeds [12]. Based on changes in protein expression in oilseeds, which contain an abundance of oils compared to starch or protein, seeds proteomics has emphasized the importance of energy and metabolism during seed development and germination. Several proteins related to oil mobilization were successfully identified in *Jatropha curcas* [6,11] and *Brassica napus* [13,14]. The tung (*Vernicia fordii* H. and *V. montana* L., previously classified as *Aleurites fordii*) tree is a multipurpose perennial plant belonging to the genus *Vernicia* of the spurge family (Euphorbiaceae). Tung is adaptable to several soil types, provided that proper drainage and aeration conditions are met [15]. Individual tung fruits typically contain multiple (usually 4–5) seeds surrounded by a thick verrucose seed coat. Tung oil, extracted from the seeds, contains 80% (mole %) α-eleostearic acid, a conjugated trienoic 18-carbon fatty acid (18:3Δ*^9cis, 11trans, 13trans^*) that imparts useful drying and blending properties to the oil [16]. Currently, tung oil is widely used in industries as a drying oil and is also used in paints, high-quality printing, plasticizers, and certain types of medicines and chemical reagents [17,18,19]. China collectively produces approximately 80,000 tons of oil per year, which represents 70%–80% of the world market [19]. However, the output of tung oil in China still will not meet the projected requirements of the international market. The ultimate objective of the tung tree breeding program in China is to create a new hybrid species or find improved varieties with enhanced oil yield, quality and resistance. 

Most of the previous research on tung oil focused on a single or a few genes that are related to the regulation of tung oil synthesis [20,21,22,23], therefore, the results of the studies have not provided a comprehensive picture of metabolism during seed development. In this study, a powerful proteomic approach was applied to study the differential protein expression in tung trees during seed development of the tung trees to provide a critical analysis that will assist in the development of new and improved breeding strategies in China.

## 2. Results

### 2.1. Tung Endosperm Cell Ultrastructure

**Figure 1:**
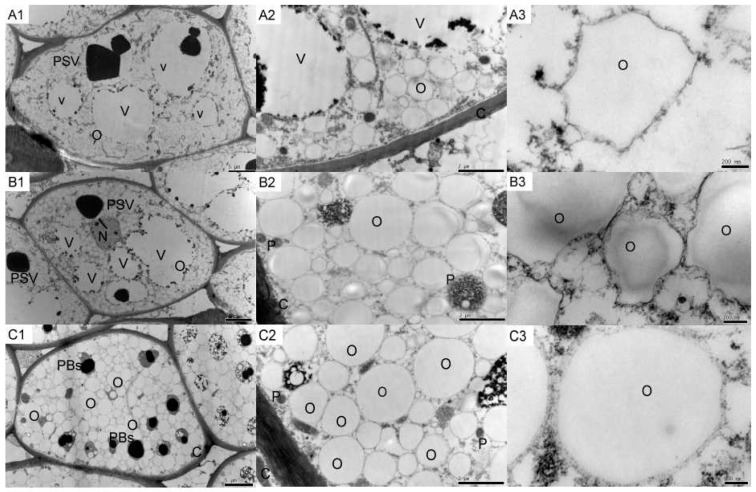
Ultrastructural observations of tung tree seeds across three development stages. Tung tree seeds prepared on: 25 August (**A**); 9 September (**B**); and 26 September (**C**). Images of: the whole tissue sample (**1**); cell sections (**2**); and single oil bodies (**3**). O, oil body; PSV, protein storage vacuole; PBs, protein bodies; P, protein particle; V, vacuole; C, cell wall; and N, nucleus.

The most obvious subcellular structures (Figure 1) were the protein storage vacuoles (PSV), vacuoles (V) and oil bodies (O). The PSV and V from the first time point (Figure 1A1) had average diameters of approximately 8 μm (±0.05, *n* = 30) and 10 μm (±0.07, *n* = 30), respectively. The diameters of the PSV and V decreased during seed growth. The structure of the PSV from the second and third time points was approximately 4 μm (±0.03, *n* = 30) (Figure 1B1) and 3 μm (±0.05, *n* = 30) (Figure 1C1), respectively. The structure of V largely disappeared during the last stage (Figure 1C1). The oil bodies were distributed throughout the entire tissue area in all three developmental stages. An obvious increase in the number and volume of the oil bodies was observed in the second and third developmental stages, as the PSV and V declined (Figure 1A2,B2,C2). In addition, the single oil bodies appeared smoother in the third developmental stage compared to the earlier time points (Figure 1A3,B3,C3).

### 2.2. Oil Content and Fatty Acid (FA) Composition

**Figure 2:**
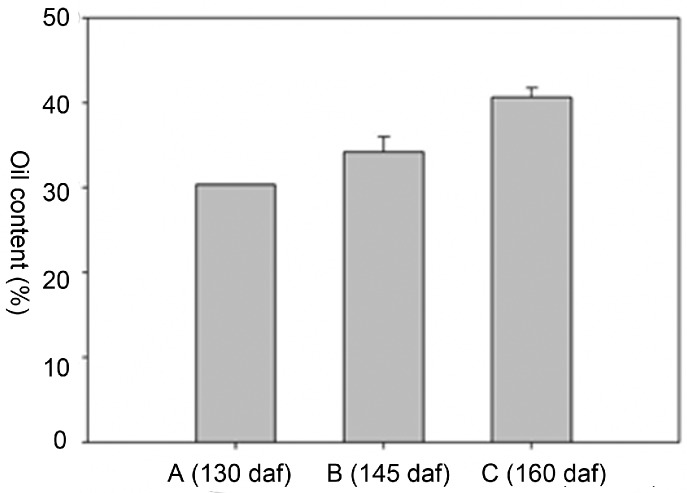
The oil content (%) in tung seeds of three developmental stages. A, B and C represent the seeds harvested on 25 August, 9 September and 26 September, respectively.

The oil content from three independent endosperm samples from each of the three different developmental stages was measured. The oil content significantly increased during seed development (Figure 2). Eleostearic acid was the most abundant fatty acid, which was followed by linoleic acid, and oleic acid. The eleostearic acid percentage of the total fatty acids increased from 72.28% to 77.5% (Table 1). Linolenic acid was undetectable at the two later time points. No significant decreases were observed for any of the other fatty acids. These results indicated that polyunsaturated fatty acids, particularly eleostearic acid, accumulate at high levels in tung seeds during all three developmental stages.

### 2.3. Comparison of the Different Protein Extraction Methods

Recalcitrant plant tissues, such as tung seeds and other plant seeds, contain many compounds which impede protein extraction. Therefore, three different methods, including trichloroacetic acid (TCA)–acetone extraction (M1), phenol extraction (M2), and TCA–acetone combined with phenol extraction (M3), were compared to identify the most suitable method for proteomic analysis (Supplementary Materials Figure S1). The protein extraction yield using M1 was the highest, followed by M2 and M3; inverse results were observed for protein quality (data not shown). The highest number of protein spots was visualized in the M1 2-DE gel followed by M3 and M2. M3 gave the best resolution of the 2-DE gels and the clearest gel background (Supplementary Materials Figure S1). These results indicated that although M3 is a complicated extraction protocol, it was the most suitable for the comprehensive proteomic analysis of tung tree seeds. 

### 2.4. Identification of Differentially Expressed Proteins in Different Functional Categories

**Figure 3:**
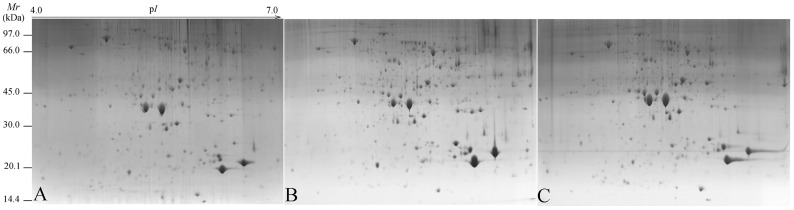
Images from the 2-DE gel maps of tung oilseeds from the three developmental stages tung tree seeds collected at three time-points: 25 August (**A**); 9 September (**B**); and 26 September (**C**).

**Figure 4:**
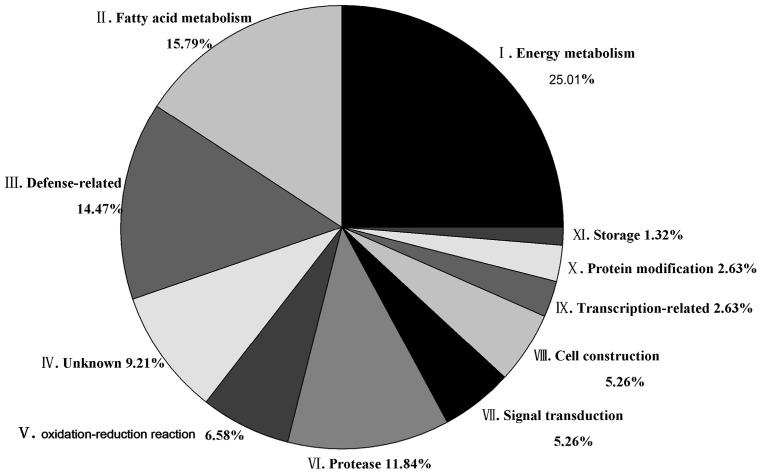
The functional proportion of the identified proteins expressed in the tung tree seeds during the three developmental stages. A total of 76 identified differential proteins were assigned to functional categories. The Roman numeral in each category corresponds to the functional category described in Supplementary Materials Table S1. The percentage represents the proportion of each category.

At least 144 protein spots changed in abundance between the three different seed developmental stages. Using MALDI-TOF/MS/MS (Figure 3), 76 protein spots were successfully identified and these protein spots were analyzed in greater detail (Supplementary Materials Figure S2, Table S1). The proteins could be sorted into eleven functional groups as follows: energy metabolism (25.01%), fatty acid metabolism (15.79%), defense-related (14.47%), protease (11.84%), unknown (9.21%), peroxidase (6.58%), signal transduction (5.26%), cell construction (5.26%), transcription-related (2.63%), protein modification (2.63%) and storage (1.32%) (Figure 4). The percentage in parentheses represents the proportion of each protein group.

#### 2.4.1. Energy Metabolism

The proteins in this group are involved in energy metabolism during seed development and included proteins with potential roles in glycolysis (S68), the tricarboxylic acid cycle (S29, S54 and S63), citric acid cycle (S45 and S56), energy-related (S11, S18 and S70) and other metabolic processes (S19, S28, S30, S33, S43, S55, S62, S72 and S74). Most of the proteins in this group, (10 out of 19 spots), were continuously up-regulated during seed development (i.e., the protein level increased in each successive time point). Only the protein of 6-phosphogluconate dehydrogenase (S18), which is a participant in the pentose phosphate pathway, was continuously down-regulated, whereas proteins S11 and S70, which are involved in other energy-related processes, were up-regulated during seed development (Supplementary Materials Table S1). In plants, glutamine synthetase (S33) (Supplementary Materials Figure S2) participates in primary metabolic processes, such as the biosynthesis of amino acids and nitrogen metabolism, and is up-regulated during tung seed development. Caffeoyl-CoA *O*-methyltransferase (S19), *S*-adenosylmethionine synthetase (S55) and UTP-glucose-1-phosphate uridylyltransferase (S72) were identified as continuously up-regulated proteins in this data set (Supplementary Materials Figure S2, Table S1), which is not surprising given their likely roles in secondary metabolic processes, such as the biosynthesis of lignin (S19) and the phenylpropanoid pathway (S55).

#### 2.4.2. Fatty Acid Metabolism

**Figure 5:**
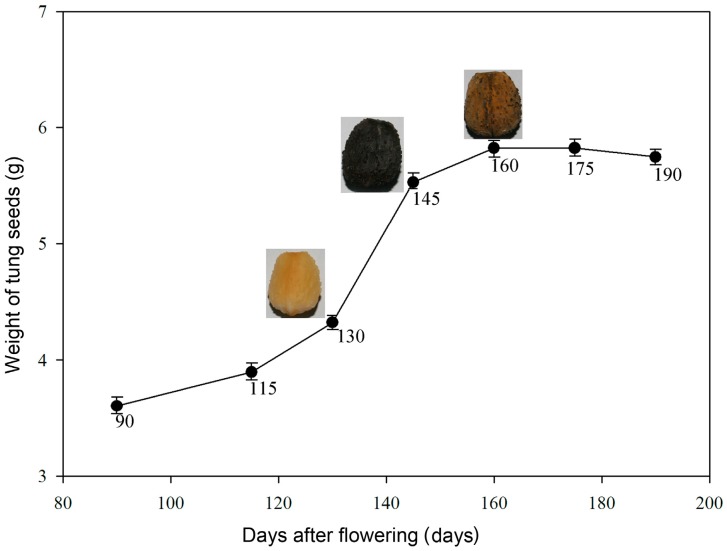
The weight of the tung seeds during development. The *x*-axis units indicate the number of days after flowering. The *y*-axis units indicate the average fresh weight (in grams) of six randomly selected seeds.

The proteins related to fatty acid metabolism in this group were mainly enzymes involved in the downstream mobilization and metabolism of oil-derived products. These enzymes function in the glyoxylate cycle, the citric acid cycle, glycolysis and the pentose phosphate pathway. Pyruvate dehydrogenase E1 component subunit alpha (S50), which participates in the citric acid cycle, showed a three-fold increase in abundance during seed development. Other up-regulated proteins included stearoyl-acyl carrier protein desaturase (S31, S57), acyl-transferase (S46), plastid 3-ketoacyl-ACP synthase (KAS) (S58) and 3-oxoacyl-[acyl-carrier-protein] reductase (S76), which likely participate in the biosynthesis of tung oil and other storage or membrane lipids in tung seeds. This result indicated that the observed increase in the accumulation of tung oil between 25 August and 26 September required increased levels of gene expression from this group. The abundance of NAD(P)-binding Rossmann-fold-containing protein (S4), esterase precursor (S17) and acetyl-CoA carboxylase (ACCase, S64) were decreased in the seeds harvested on 26 September compared with the seeds of 25 August (Figure 5). These data, particularly for ACCase, suggested that fatty acid synthesis begins to decline as the seeds approach complete maturation. Noticeably, the fatty acid metabolism group shares some proteins with the energy metabolism group, including malate dehydrogenase (S63), which plays an important role in the glyoxylate cycle.

#### 2.4.3. Defense-Related

The proteins in this group are mainly involved in the stress-response (S3, S69), protection (S13, S14, S32, S34, S47) and detoxification (S23, S37, S59, S61). Most of the proteins in this group were up-regulated and their likely functions involved responses to abiotic stresses given their roles in cold, heat, salt and drought tolerance in many other plant species [24]. Nucleoside diphosphate kinase 2 (NDPK2) (S3), which is a member of NDP kinases family, enhances salt stress tolerance [25]. Glycine-rich proteins (GRP) (S69) improve tolerance against freezing stresses [26]. Defense-related proteins, similar to GRP (S10), are associated with signal transduction pathway. Heat shock proteins (HSP) (S13, S14, S32, S34, and S47) were also up-regulated, likely to assist in proper protein folding and to guard against the deleterious effects of the high temperatures encountered during the summer months as tung seeds mature [27]. Superoxide dismutase (SOD) (S23 and S37) may act as a first line of defense against reactive oxygen species (ROS), a process in which MnSOD (S37) is the principal antioxidant enzyme [28]. Cysteine protease inhibitor (S59) and alcohol dehydrogenase (S61) were up-regulated during seed development and likely participate in detoxification pathways. Other functional categories included protein modification, storage, peroxidase, signal transduction, cell construction, transcription-related, and proteins with unknown functions (Supplementary Materials Table S1).

## 3. Discussion

### 3.1. Efficient Extraction Methods for Tung Tree Seed Proteins

Isaacson et al. [29] recently compared the following two basic protocols for plant proteomics protein preparation: one is based on TCA–acetone extraction and the second relies on phenol precipitation. As the most commonly used protein extraction technique, TCA–acetone extraction results in protein pellets that are hard to re-solubilize and always retains some insoluble material, which results in protein losses. However, a high number of plant samples can be processed using TCA–acetone extraction. This method effectively inhibits proteases as well as phenoloxidases and peroxidases [30]. Phenol extraction protocols were first reported by Hurkman and Tanaka [31]. Phenol interacts with proteins through hydrogen bonding and causes proteins to become denatured and soluble in the organic phase. Phenol also acts as a dissociating agent that decreases molecular interactions between proteins and other materials [32]. Previous studies have indicated that use of phenol results in less streaking on both the horizontal and vertical dimensions of two-dimensional protein gels compared to samples prepared using TCA–acetone [30,33].

However, both strategies are usually applied to relatively “easy” tissues, such as etiolated shoots and young root tips [29]. An efficient strategy for protein extraction from recalcitrant plant tissues (e.g., seeds) using a combination of TCA–acetone and phenol extraction was presented here, which resulted in high-quality protein samples according to definitions presented by Wang et al. [34]. In this report, three protein extraction methods for tung tree seeds were compared with respect to the protein yield, quality and resolution of the 2-DE gel. The protein yield obtained by the combination of TCA–acetone and phenol preparation (M3) was relatively low, but resulted in the best protein quality and gel resolution (Supplementary Materials Figure S1). Thus, method M3 was deemed the most suitable for the proteomic analysis of the tung tree seeds.

### 3.2. The Relationship between Oil Bodies and Oil Content in Seeds During Developmental Stages

Previous studies have indicated that there is a close relationship between oil content and oil bodies in various plant species. The differences in oil body abundance in high oil versus low oil cultivars of *Brassica napus* indicated that larger (and presumably fewer) oil bodies exist in the lower oil content cultivars [35]. Large oil bodies exist in high oil content cultivars of maize [36], which is influenced, at least in part, by the level of seed oleosin proteins. Oil bodies develop during embryo development in higher-oil sunflower cultivars, and then stop developing before the complete growth of the embryo is achieved [37].

In this study, the changes in the cellular ultrastructure, oil body number, and oil content during development of tung seed endosperm were analyzed. According to the results, the oil content in the seeds increased from 25 August to 26 September. In the 25 August seeds, the average oil content was 30.3%, and increased to 34.2% and 40.6%, during the second and third time points, respectively (Figure 2). The ultrastructural observations of the endosperm cells showed that the volumes of the oil bodies decreased, whereas the number of oil bodies increased through progressive developmental stages (Figure 1, Supplementary Materials Figure S3).

### 3.3. Seed Reserve Synthesis during Development

Seed reserves are mainly accumulated during seed development for the next generation of germination and seedling growth. The most important reserves are oil, proteins and starches, and the seeds of different species can be categorized according to the relative amounts of these three metabolites. In the seeds of the tung tree, oil is the most abundant reserve. From 25 August to 26 September, the oil content in the seeds increased substantially (Figure 2).

At the protein level, malate dehydrogenase (S29 and S63) was down-regulated from 25 August to 9 September, whereas the enzymes related to oil synthesis (S31, S40, S57, S58 and S64) were up-regulated across these the three developmental stages (Supplementary Materials Table S1, Figure 5), which correlated with the increase in oil content and oil body abundance in the 26 September seeds (Figure 3 and Figure S3). These results provide strong molecular-level support for the notion that oil, as the primary reserve in tung seeds, actively accumulates throughout a large portion of seed development and that much of the gene expression in these organs is dedicated to the pathways necessary for oil biosynthesis.

### 3.4. Some Enzymes Were Undetectable during Seed Development

Lipid metabolism in plant seeds is complex and requires over 100 enzymatic reactions and 600 proteins [38]. An oil body consists of a neutral lipid core enclosed by a membrane lipid monolayer that is primarily coated with structural proteins, such as oleosins and caleosin [39,40]. It was surprising that oleosins, the most abundant oil body-associated proteins, were not found in this study. This may be because the predicted isoelectric point for oleosins is 9.7 and the pH gradient strips (IPGs) used in present study ranged from pH 4 to 7. Consequently, all the oleosins may have been lost during the first dimension IEF [41]. Caleosin, which is another important protein related to oil bodies, is a Ca^2+^-binding oil body surface protein that participates in oil-vacuole interactions that affect the breakdown of oil bodies during germination [8,42]. Storage proteins accumulate during seed development, particularly at the late stages of development [43]. However, few storage proteins were identified other than a nutrient reservoir protein (S67), which was found to be continuously up-regulated in all three developmental stages (Supplementary Materials Figure S2) and indicated its role in nutrient deposition during seed development. 

This data set represents a significant step forward in oilseed proteomics; however, we identified fewer proteins than expected. A significant limitation to MS identification could be the lack of currently available genomic resources for the tung tree. In the study, the tung tree proteins were identified using homology to the sequences of other oil or woody plants including *Arabidopsis* and *Ricinus communis*. Nonetheless, the results shown here provide a clear basis to better understand the interplay between different metabolic pathways in developing oilseeds and will help to guide the rational design of future experiments. 

## 4. Materials and methods

### 4.1. Materials

The seeds were harvested approximately 130 days (25 August), 145 days (9 September) and 160 days (26 September) after flowering, and were marked A, B and C in this paper, respectively (Figure 5). Tung tree fruits were harvested from trees in the State-owned Dongfanghong Forest Farms located in Jinhua City (E119°14’–E120°46’, N28°32’–N29°41’), Zhejiang Province, China. The seeds were immediately removed from the fruit, and the seed fractions were stored at −80 °C after quick-freezing in liquid nitrogen and used for protein extraction and two-dimensional gel electrophoresis (2-DE). Other seed fractions were stored at −4 °C, and used for oil content and fatty acid composition analysis.

### 4.2. Tissue Preparation for Transmission Electron Microscopy

For the ultrastructural observation studies, seed endosperm tissues were cut from the seeds as blocks (2 mm length × 1 mm width × 1 mm height). The tissue blocks were fixed in 2.5% glutaraldehyde in 100 mM phosphate buffer (pH 7.0) for 4 h at room temperature, and then washed with 100 mM phosphate buffer (pH 7.0). Subsequently, the blocks were treated with 1% osmic acid (OsO_4_) and rinsed with phosphate buffer (3 × 15 min) and then dehydrated using a graded series of ethanol treatments (50%, 70%, 85%, 95%, and 100% *v*/*v*). The infiltration process was completed using a graded ethanol/Epon/Spurr’s epoxy resin series, followed by embedding the blocks in 100% (*w*/*v*) Spurr’s epoxy resin and polymerization at 60 °C for 24 h. The samples were cut into thin sections (70 nm) using an ultramicrotome (Leica MZ6, LeiMicrosystems, Wetzlar, Germany), and then collected onto copper grids, and poststained with saturated uranyl acetate and 0.4% lead citrate. After the sections were rinsed with dH_2_O (6 × 15 s), they were viewed with a H-7650 transmission electron microscope (Hitachi company, Koka, Japan).

### 4.3. Oil Content and Fatty Acid Composition Analysis

Endosperm tissues collected at different developmental stages were milled into a fine powder and the oil content was analyzed using Soxhlet extraction. Fatty acid composition analysis was performed according to the methods described by Yang [8].

### 4.4. Protein Extraction, Two-Dimensional Gel Electrophoresis and Data Analysis

Effective separation and identification of proteins depends upon efficient protein isolation methodology. TCA–acetone extraction [44], phenol extraction [45] and TCA–acetone/phenol extraction [46] with a minimal modification were used to prepare the protein samples of tung seeds and the effects of these extraction methods were compared by 2-DE and sodium dodecyl sulfate polyacrylamide gel electrophoresis (SDS-PAGE) in pre-experiment. The results of pre-experiment indicated that TCA–acetone combined with phenol extraction method was suitable for the protein extraction from tung seeds [47]. The detailed protocols of TCA–acetone combined with phenol extraction method for protein extraction and 2-DE in this study are as follows.

Seed endosperms were ground into a fine power under liquid nitrogen. During grinding, 10% (*w*/*w*) polyvinyl-polypyrrolidone (PVPP) was added. Dry powdered tissue (0.5 g/sample) was transferred into centrifugation tubes, and suspended in 2 mL of extraction buffer I [10% (*w*/*v*) TCA, 0.07 (*v*/*v*) β-mercaptoethanol (β-ME) in cold acetone and 18 μL phenylmethylsulfonyl fluoride (PMSF, 200 mM)], for 2 h at −20 °C. After centrifugation at 12,000 rpm for 15 min at 4 °C, the supernatants were removed and the precipitated pellets were washed in 1 mL cold acetone containing 0.07% β-ME for 5 min. After centrifugation, the pellets were rinsed again with 0.1 mM ammonium acetate in methanol for 5 min. Then, the pellets were homogenized using Tris-saturated phenol and an equal volume of extraction buffer II [500 mM Tris-HCl (pH 8.65), 50 mM ethylenediaminetetraacetic (EDTA), 2% (*v*/*v*) 100 mM KCl] at −20 °C for 2 h. The phenol phase was collected, and protein was precipitated overnight at −20 °C after adding 0.1 mM ammonium acetate in methanol. After centrifugation, the proteins were rinsed once each in 0.1 mM ammonium acetate in methanol and 100% cold acetone, respectively. The pellets were vacuum-dried for 1 h and stored at −80 °C for later use.

The dried protein pellets were dissolved in solution buffer containing 7 M urea, 2 M thiourea, 4% 3-[(3-cholamidopropyl)-dimethylammonio]-1-propane sulfonate (CHAPS), 65 mM dithiothreitol (DTT), and 2% (*v*/*v*) ampholine pH 3–10 (GE Healthcare Bio-Science, UK Ltd., Buckinghamshire, UK). After centrifugation at 12,000 rpm for 15 min, the supernatant was collected for protein quantification.

The immobilized pH gradient strips (IPGs, pH 4–7, 24 cm, GE Healthcare Bio-Science, UK Ltd.) were used in two-dimensional gel electrophoresis on the Multiphor^TM^ II Electrophoresis System (GE Healthcare Bio-Science, UK Ltd.). A total of 400 μL sample containing 850 μg proteins were loaded in IPGs for rehydration at 50 V and 20 °C constant temperature. Isoelectric focusing (IEF) was performed at 500 V for 30 min, 1000 V for 1.5 h and 8000 V for 4 h, and finished at 70,000 Vh, all at 50 μA/strip. After the IEF run, the IPGs were equilibrated in equilibration buffer [6 M urea, 2% (*w*/*v*) SDS, 20% (*w*/*v*) glycerol, 375 mM tris-HCl (pH 8.8) and 2% (*w*/*v*) DTT] at room temperature for 15 min. The second equilibration was carried out in the same buffer, except that DTT was replaced with 2.5% (*w*/*v*) iodoacetamide. Second-dimension protein separation and visualization was achieved by 12.5% SDS-PAGE electrophoresis and post-staining with 0.1% Coomassie Brilliant Blue (CBB) R-250.

The images of 2-DE gels were analyzed using Image Master 2D Platinum Version 5.0 Analysis Software (GE Healthcare Bio-Science, UK Ltd.). Only the protein spot abundances which changed more than 1.2-fold across the three different developmental stages. 

### 4.5. In-Gel Protein Digestion and MALDI-TOF/MS Analysis

Protein digestion and MALDI-TOF/MS analyses were performed as follows. Each protein gel piece was destained with 100 mM NH_4_HCO_3_ in 30% (*v*/*v*) acetonitrile (ACN) for 2 h at 40 °C. The gel pieces were minced, lyophilized and digested in 25 mM NH_4_HCO_3_ with 10 ng sequencing-grade modified trypsin (Promega, Madison, WI, USA) at 37 °C overnight. After digestion, peptides were extracted by three washes with 0.1% trifluoroacetic acid (TFA) in 60% ACN. The peptides were desalted by ZipTipC-18 pipet tips (Millipore, Bedford, MA, USA) according to the product manual. Tryptic peptide samples were analyzed using a 4800 Plus MALDI TOF/TOFTM Analyzer (Applied Biosystems, Foster, CA, USA).

## Figures and Tables

**Table 1:** Changes in the fatty acid composition (%) of tung tree seed oil in the three developmental stages.

Time	Fatty Acid					
	Palmitic Acid	Stearic Acid	Oleic Acid	Linoleic Acid	Linolenic Acid	Eleostearic Acid
8.25 (A)	3.74 ± 0.02	2.75 ± 0.03	7.42 ± 0.08	13.82 ± 0.12	0.21 ± 0.15	72.28 ± 0.06
9.9 (B)	2.50 ± 0.05	1.95 ± 0.01	7.99 ± 0.06	9.11 ± 0.01	-	77.47 ± 0.05
9.26 (C)	2.35 ± 0.06	2.17 ± 0.02	8.03 ± 0.08	7.85 ± 0.05	-	77.49 ± 0.02

The content of each fatty acid was calculated as the percentage of the total measured fatty acids. The “-” denotes that the fatty acid data were undetectable. The values are the means of three biological replicates (±SD).

## Supplementary Materials

Supplementary materials can be accessed at: http://www.mdpi.com/1420-3049/21/11/1468/s1.
