# Supplementary material for: Proteomic Analysis of Tung Tree (Vernicia fordii) Oilseeds during the Developmental Stages
Source: Molecules. 2016 Nov 8;21(11):1486. doi: 10.3390/molecules21111486 (PMC6273751; doi:10.3390/molecules21111486)
Supplement: Supplementary file 1 [file molecules-21-01486-s001.pdf]

Supplementary Materials: Proteomic Analysis of Tung Tree (*Vernicia fordii*) Oilseeds during the Developmental Stages

Zhiyong Zhan, Yicun Chen, Jay Shockey, Xiaojiao Han and Yangdong Wang

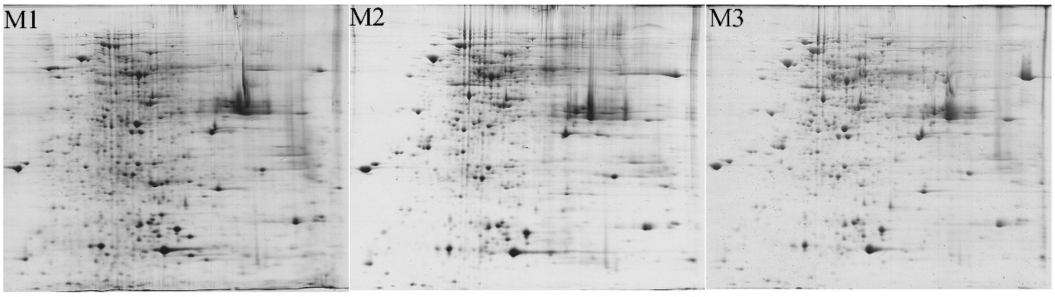

Figure S1. Images from 2-DE gel samples prepared using different protein extraction methodsin three time-point. (**M1**) TCA-acetone extraction; (**M2**) Phenol extraction; (**M3**) TCA-acetone combined with phenol extraction.

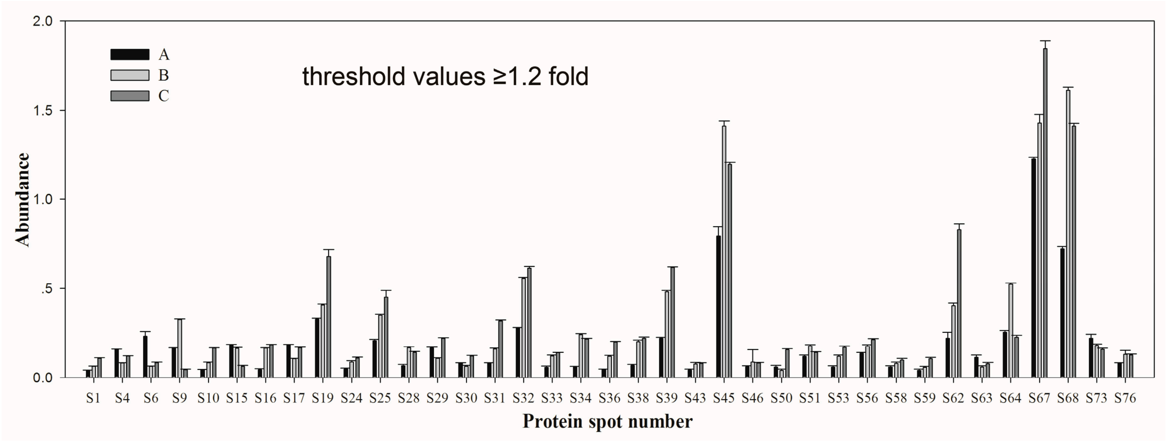

Figure S2. Abundance of 37 differentially expressed proteins. For each spot, each value is the mean of three biological replicates (±SD). Spot numbers correspond to the numbers described in Table 1. A, B and C represent the seeds harvested on 25 August, 9 September and 26 September.

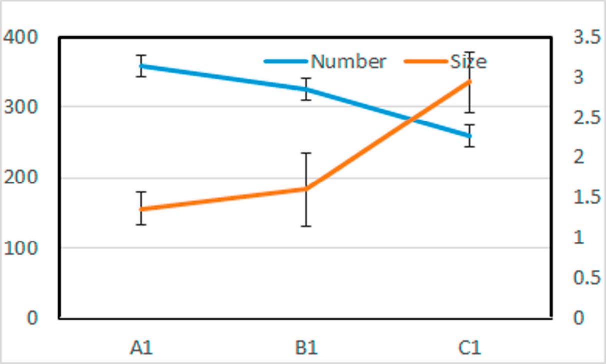

Figure S3. The volumes of oil bodies decreased and the numbers increased through progressive developmental stages. A1, B1 and C1 represent the seeds harvested on 25 August, 9 September and 26 September.

Table S1. Tung tree seed proteins identified by MALDI-TOF MS.

SN2 [1]	Protein Name	Accession Number.	Homology to Species	Sequences	M*r*/p*I*	PS3 [2]	PC4 [3]	Comparison [4]		
								α	β	γ
	Energy metabolism									
S11	Rubisco subunit binding-protein alpha subunit	gi∣255587664	*Ricinus communis*	AIELPDPMENAGAALIR	53,280.5/5.25	192	100	U	U	U
S18	6-phosphogluconate dehydrogenase	gi∣255537671	*Ricinus communis*	DLFGAHTYER	54,510.8/6.25	190	100	D	D	-
S19	Caffeoyl-CoA *O*-methyltransferase	gi∣120561153	*Brassica rapa*	VEISQISVGDGVTLCR	19,595.2/4.64	85	99.6	U	U	U
S28	Lactoylgutathione lyase	gi∣255554865	*Ricinus communis*	DPDGYIFELIQR	31,641.3/7.63	295	100	U	D	U
S29	Malate dehydrogenase, cytoliasmic	gi∣225438145	*Vitis vinifera*	VLVTGAAGQIGYALVPMIAR	35,881.4/6.18	107	99.9	D	U	U
S30	Cinnamoyl-CoA reductase	gi∣255544904	*Ricinus communis*	DVANAHIQAFEIPSASGR	45,052/6.39	76	97.5	D	U	U
S33	Glutamine synthetase	gi∣2213877	*Hevea brasiliensis*	GNNILVMCDAYTPAGEPIPTNKR	39,503.7/5.81	241	100	U	U	U
S43	5-methyltetrahydropteroyltriglutamate-homocysteine methyltransferase	gi∣255549601	*Ricinus communis*	FLFAGVVDGR	90,473.4/6.45	158	100	U	U	-
S45	Enolase	gi∣356559925	*Glycine max*	AAVPSGASTGVYEALELR	48,230.8/6.06	234	100	U	D	U
S54	Succinate semialdehyde dehydrogenase	gi∣255577875	*Ricinus communis*	EEVFGPVAPILR	65,727.4/8.84	97	99.9	U	U	D
S55	*S*-adenosylmethionine synthase	gi∣356565143	*Glycine max*	FVIGGPHGDAGLTGR	43,448.9/5.65	120	100	U	U	U
S56	Pyruvate dehydrogenase	gi∣255557267	*Ricinus communis*	SENPVILFEHVLLYNLK	44,901.7/6.09	199	100	U	U	U
S62	Cytosolic, 3-phosphoglycerate kinase	gi∣28172915	*Zea mays*	LASVADLYVNDAFGTAHR	31,662.8/5.01	207	100	U	U	U
S63	Malate dehydrogenase, putative	gi∣255541140	*Ricinus communis*	VLVTGAAGQIGYAIVPMIAR	36,058.6/6.19	112	100	D	U	D
S68	Triosephosphate isomerase, cytosolic	gi∣226495391	*Zea mays*	VIACVGETLEQR	27,236.1/5.52	167	100	U	D	U
S70	Ribulose-1,5-bisphosphate carboxylase/oxygenase	gi∣18140587	*Laplacea fruticosa*	VTPQPGVPPEEAGAAVAAESSTGTWTTVWTDGLTSLDR	52,058.2/6.34	604	100	D	U	U
S71	Legumin A precursor	gi∣255567248	*Ricinus communis*	AMPIEVVANAFQVSVDEA	57,872.6/8.03	81	99.1	D	D	D
S72	UTP-glucose-1-phosphate uridylytransferase	gi∣255571289	*Ricinus communis*	VQLLEIAQVPDEHVNEFK	51,770.4/5.71	234	100	U	U	-
S74	Diaminopimelate epimerase	gi∣255584553	*Ricinus communis*	YQGLGNDFILVDNR	40,540.1/6.07	152	100	U	U	U
	Fatty acid metabolism									
S4	NAD(P)-binding rossmann-fold-containing protein	gi∣18404496	*Arabidopsis thaliana*	KAEQYLADSGIPYTIIR	34,972.2/8.37	276	100	D	U	D
S9	Methionine adenosyltransferase	gi∣75297911	*Arabidopsis thaliana*	FVIGGPHGDAGLTGR	43,333.8/5.67	74	95.3	U	D	D
S17	Esterase precursor	gi∣255560956	*Ricinus communis*	ALYTFDIGQNDLSVGFR	46,194.2/6.6	513	100	D	U	D
S31	Stearoy-acyl-carrier protein desaturase	gi∣110741382	*Arabidopsis thaliana*	TENNPYLGFIYTSFQER	32,330.3/5.94	191	100	U	U	U
S40	Plastid-lipid-Associated protein	gi∣2632088	*Nicotiana tabacum*	QLVDSFYGTNR	28,385.4/4.83	90	99.8	U	U	D
S46	HXXXD-type acyl-transferase-like protein	gi∣15229017	*Arabidopsis thaliana*	RLVELVTDGFMR	48,315.9/5.51	74	96.2	U	U	-
S50	Pyruvate dehydrogenase E1 component subunit alpha-like	gi∣356520231	*Glycine max*	SQLLENVFADPK	48,315.1/6.41	147	100	D	U	U
S51	3-hydroxybutyryl-CoA dehydratase	gi∣255549046	*Ricinus communis*	NDFAEGVR	45,616.1/5.97	142	100	U	U	D
S57	Stearoyl-acyl carrier protein desaturase	gi∣3355632	*Linum usitatissimum*	TENNPYLGFIYTSFQER	44,999.7/5.81	220	100	U	U	U
S58	Plastid 3-ketoacyl-ACP synthase Ⅰ	gi∣74475410	*Cuphea avigera var. pulcherrima*	GAPIIAEYLGGAINCDAYHMTDPR	49,912.2/6.86	88	99.8	U	U	U
S64	Acetyl-CoA carboxylase	gi∣347662503		MILAGYEHDIDK	259,206.8/5.99	71	91.2	U	D	D
S76	3-oxoacyl-[acyl-carrier-protein] reductase	gi∣356540173	*Glycine max*	ILETIPLGR	33,862.7/9.17	302	100	U	U	-
	Defense-related									
S3	Nucleoside diphosphate kinase 2	gi∣145712785	*Arabidopsis lyrata subsp. petraea*	GLVGEIISR	22,522.7/9.27	78	98.5	D	-	U

S13	Heat shock protein, putative	gi|255554571	*Ricinus communis*	AVITVPAYFNDAQR	71,304/6.1	129	100	U	U	-
S14	Heat shock protein, putative	gi|255575054	*Ricinus communis*	EQVFSTYSDNQPGVLIQV YEGER	71,470.3/5.07	299	100	U	U	-
S23	Chloroplast Cu/Zn superoxide dismutase	gi|86920323	*Arabidopsis thaliana*	AFVVHELEDDLGKGGHE LSLTTGNAGGR	6977.5/4.41	199	100	U	-	D
S32	17.5KDa class Ⅰ heat shock protein-like	gi|356521398	*Glycine max*	ETPEAHVFEADIPGLKK	17,534.9/5.33	200	100	U	U	U
S34	18.8KDa class Ⅱ heat shock protein-like	gi|225429618	*Vitis vinifera*	EYPNAYVFLVDMPGLK	18,463.6/8.46	87	99.7	U	D	U
S37	Manganese superoxide dismutase	gi|9929159	*Digitalis lanata*	HHQAYITNYNK	24,978.9/8.01	135	100	U	U	U
S47	Heat shock 70KDa protein	gi|357120971	*Brachypodium distachyon*	SKFESLVNNLIER	121,337.9/5.4	124	100	U	U	D
S59	Cysteine protease inhibitor	gi|1638842	*Ricinus communis*	QVVAGTLHHLTIEAIEAGK	23,289.8/6.17	72	93.9	U	U	U
S61	Alcohol dehydrogenase	gi|255570416	*Ricinus communis*	GQSVAIFGLGAVGLAAA EGAR	42,038.1/5.91	207	100	U	U	-
S69	Glycine-rich protein	gi|171451986	*Bruguiera gymnorhiza*	NITVNEAQSR	17,036.1/9.81	83	99.4	D	D	U
	Unknown									
S1	Hypothetical protein Osl_34099	gi|218184783	*Oryza sativa Indica Group*	AAAGSSIHTMLTGR	62,243.7/5.83	84	99.6	U	U	U
S7	Os01g0964400	gi|297720793	*Oryza sativa Japonica Group*	AGFAGDDAPR	24,702.4/6.08	172	100	U	U	U
S8	Conserved hypothetical protein	gi|255568303	*Ricinus communis*	ELVLGQDQPR	60,659.1/5.89	89	99.8	U	U	D
S27	Conserved hypothetical protein	gi|255568303	*Ricinus communis*	EQPSLEEISKLR	60,659.1/5.89	79	98.6	D	D	-
S38	OSIGBa0140J09.5	gi|116310837	*Oryza sativa Indica Group*	DLCEDFGIK	160,858.4/9.3	73	95.0	U	U	U
S39	Hypothetical protein SELMODRAFT_410993	gi|302773530	*Selaginella moellendorffii*	ELELLTPER	37,543.8/6.34	80	98.8	U	U	U
S41	Predicted protein	gi|224138130	*Populus trichocarpa*	DLFEILMDENR	39,787.6/5.77	182	100	U	U	D
	Peroxidase									
S5	Peroxiredoxin, putative	gi|255556526	*Ricinus communis*	LSFLYPASTGR	24,321.4/5.64	133	100	U	U	U
S24	Peroxiredoxin	gi|300078580	*Jatropha curcas*	SYGVLIPDQGIALR	25,054.9/8.37	505	100	U	U	U
S26	Peroxiredoxin	gi|255556526	*Ricinus communis*	LSFLYPASTGR	24,321.4/5.64	174	100	D	D	-
S60	Peroxiredoxin	gi|2555575353	*Arabidopsis thaliana*	EQPSLEEISKLR	23,943.7/7.63	186	100	U	U	-
S66	Glutathione peroxidase	gi|255537447	*Ricinus communis*	FLVDKDGNVVDR	18,717.5/6.58	183	100	U	U	U
	Protease									
S12	Proteasome subunit alpha type, putative	gi|255538698	*Ricinus communis*	NQYDTDVTTWSPAGR	30,638.1/4.89	395	100	D	D	U
S20	Proteasome subunit alpha type-5-A	gi|15220961	*Arabidopsis thaliana*	FSYGEPMTVESTTQALC DLALR	26,102/4.7	342	100	U	U	U
S25	Proteasome subunit	gi|255541320	*Ricinus communis*	GCVYTYDAVGSYER	24,880.2/6.08	216	100	U	U	U
S35	26S protease regulatory subunit 6A	gi|195635679	*Zea mays*	DSYLILDTLPSEYDSR	47,886.6/4.98	102	99.9	U	U	-
S48	Aspartic proteinase precursor	gi|255578112	*Ricinus communis*	EPVFSFWFNR	56,595.9/5.19	91	99.9	U	-	D
S49	26S protease regulatory subunit 6a, putative	gi|255542742	*Ricinus communis*	DSYLILDTLPSEYDSR	48,271/5.05	201	100	U	U	D
S53	Peptidase	gi|255571742	*Ricinus communis*	QPGVLQVVPSR	13,853.3/8.04	96	99.9	U	U	U
S52	Proteasome subunit beta type	gi|255558626	*Ricinus communis*	LFLGLSGLATDAQTLYQR	23,126.6/5.17	598	100	D	D	U
S65	Nascent polypeptide associated complex alpha subunit	gi|255569201	*Ricinus communis*	IEDLSSQLQTQAAEQFK	14,221.1/4.24	308	100	D	D	D
	Signal transduction									
S2	Nucleoside diphosphate kinase	gi|255537805	*Ricinus communis*	TFIAIKPDGVQR	25,627/9.16	143	100	U	U	D
S10	*Glycine-rich RNA-binding protein*	gi|255571692	*Arabidopsis thaliana*	CDEPAMPKTMCLR	16,562.6/7.79	193	100	U	U	U
S44	Profilin	gi|30841324	*Gossypium hirsutum*	FMVIQGEPGAVIR	14,924.4/5.38	128	100	D	-	U
S75	Nucleoside diphosphate kinase	gi|284433792	*Jatropha curcas*	IIGATNPAESAPGTIR	16,339.5/6.32	499	100	D	D	U

	Cell construction									
S6	Actin	gi∣50660331	*Vitis vinifera*	NYELPDGQVITIGAER	19,185.8/4.86	605	100	D	U	D
S15	Actin-like protein	gi∣294884347	*Populus tremula x Populus alba*	NYELPDGQVITIGAER	16,084.2/4.78	559	100	D	D	D
S16	Myosin class II heavy chain (ISS)	gi∣308801757	*Ostreococcus tauri*	ELEHQMSLLEEQR	30,4318.4/5.0	79	98.6	U	U	U
S36	Actin	gi∣296881978	*Jatropha curcas*	NYELPDGQVITIGAER	41,973.1/5.31	767	100	U	U	U
	Transcription-related									
S21	Peptidyl-prolyl cis-trans isomerase	gi∣359480227	*Vitis vinifera*	CFFDVDIGGEPVGR	23,214.2/8.25	123	100	U	-	D
S22	Nuclear transport factor 2 isoform 1	gi∣225425388	*Vitis vinifera*	AFVEHYYSTFDANR	13,699.7/5.67	119	100	U	U	U
	Protein modification									
S42	Ubiquitin 1, putative	gi∣255572315	*Ricinus communis*	TLTGKEIEIDIEPTDTIDR	13,525.3/6.6	639	100	D	D	-
S73	Ubiquitin	gi∣111218906	*Arabidopsis thaliana*	IQDKEGIPPDQQR	24,638.2/6.4	382	100	D	D	D
	Storage									
S67	Nutrient reservoir	gi∣255570801	*Ricinus communis*	REGEDWFLLQDSK	52,414.5/6.4	80	98.9	U	U	U

Proteins identified in the table with a false discovery rate (FDR) < 0.05. M*r*/p*I* indicates the protein molecular weight and isoelectric point. Accession no. is the NCBI database identifier. [1]. (Spot no.) indicates the different expression protein spots which selected by the software during the process of 2-DE gels analysis. [2] and [3] are the thresholds. Generally, PS (Protein Score) > 60 and PC (Protein Score C.I. %) > 95 mean that the protein identification analysis results from MALDI TOF/MS were credible. [4]. U and D represent up-regulated and down-regulated protein spots, respectively. The protein spots marked by "-" indicated these spots were only undetectable in the group by the methods used in this report. (α) the sample from 25 August was compared with 9 September period (β) the sample of 9 September compared with 26 September. (γ) the sample of 25 August compared with 26 September.
